# Classification of diffraction patterns using a convolutional neural network in single-particle-imaging experiments performed at X-ray free-electron lasers

**DOI:** 10.1107/S1600576722002667

**Published:** 2022-04-22

**Authors:** Dameli Assalauova, Alexandr Ignatenko, Fabian Isensee, Darya Trofimova, Ivan A. Vartanyants

**Affiliations:** a Deutsches Elektronen-Synchrotron DESY, Notkestraße 85, 22607 Hamburg, Germany; b Applied Computer Vision Lab, Helmholtz Imaging, German Cancer Research Center (DKFZ), Im Neuenheimer Feld 280, 69120 Heidelberg, Germany; c Division of Medical Image Computing, German Cancer Research Center (DKFZ), Im Neuenheimer Feld 280, 69120 Heidelberg, Germany

**Keywords:** convolutional neural networks, single-particle imaging, classification of diffraction patterns, X-ray free-electron lasers

## Abstract

Single particle imaging (SPI) at X-ray free-electron lasers is particularly well suited to determining the 3D structure of particles at room temperature. For a successful reconstruction, diffraction patterns originating from a single hit must be isolated from a large number of acquired patterns. It is proposed that this task could be formulated as an image-classification problem and solved using convolutional neural network (CNN) architectures. Two CNN configurations are developed: one that maximizes the F1 score and one that emphasizes high recall. The CNNs are also combined with expectation-maximization (EM) selection as well as size filtering. It is observed that the CNN selections have lower contrast in power spectral density functions relative to the EM selection used in previous work. However, the reconstruction of the CNN-based selections gives similar results. Introducing CNNs into SPI experiments allows the reconstruction pipeline to be streamlined, enables researchers to classify patterns on the fly, and, as a consequence, enables them to tightly control the duration of their experiments. Incorporating non-standard artificial-intelligence-based solutions into an existing SPI analysis workflow may be beneficial for the future development of SPI experiments.

## Abstract 2

A convolutional neural network is applied for the single-hit diffraction-pattern classification step in single-particle-imaging experiments at X-ray free-electron lasers. This approach can be employed not only after the experiment but, importantly, also during an experiment and can significantly reduce the size of data storage for further analysis stages.

## 1. Introduction

Artificial intelligence (AI) and machine learning methods are rapidly becoming an important tool in physics research. We have witnessed an increased interest in these approaches, especially during recent years. This is also related to the large amount of data collected nowadays in experiments not only in particle physics but also in astronomy and X-ray physics. For example, petabytes of data can easily be collected within just a few days at a single beamline of the megahertz European X-ray Free-Electron Laser (Decking *et al.*, 2020 ▸). Machine learning approaches can help us to use this enormous quantity of data effectively.

One of the flagship experiments at X-ray free-electron lasers (XFELs) is single particle imaging (SPI). In these experiments, single biological particles such as viruses or protein complexes are injected into the intense femtosecond XFEL beam in their native environment, and diffraction patterns are collected before particles are disintegrated as a result of Coulomb explosion (Neutze *et al.*, 2000 ▸). By collecting a sufficient number of diffraction patterns originating from reproducible biological samples at different orientations, the full 3D diffracted intensity may be obtained and then, applying phase-retrieval techniques, a high-resolution image of the biological sample may be reconstructed (Gaffney & Chapman, 2007 ▸). Despite being well defined, the problem of obtaining high-resolution images of single biological particles at an XFEL is still far from being solved. In order to determine the best strategies to push SPI to higher resolution, the SPI consortium was formed at the Linac Coherent Light Source (LCLS) at SLAC National Accelerator Laboratory (Stanford, USA) (Aquila *et al.*, 2015 ▸).

**Figure 1:**
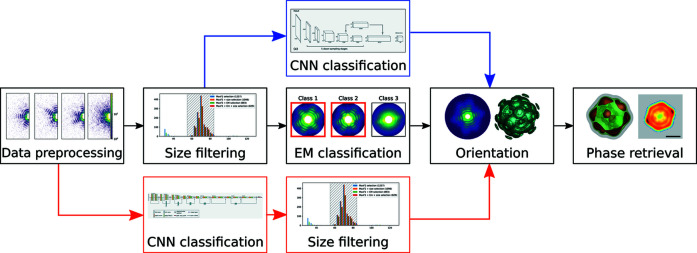
SPI workflow. Black arrows indicate the typical steps in SPI data analysis (Assalauova *et al.*, 2020 ▸). Blue arrows show the implementation of CNN-based single-hit diffraction-pattern classification (Ignatenko *et al.*, 2021 ▸). Red arrows show the modified workflow for CNN-based classification prior to the particle size filtering step (this work).

In the framework of this consortium, several strategies for data analysis were developed. Typical SPI data analysis comprises a few sequential steps from the raw detector images to the 3D reconstructed particle structure (see Fig. 1 ▸). This workflow consists of the following steps: initial pre-processing of diffraction patterns, particle size filtering, single-hit diffraction-pattern classification, orientation determination and obtaining the 3D intensity map of the particle, and, finally, phase retrieval and reconstruction of the 3D electron density of the biological sample (Gaffney & Chapman, 2007 ▸; Rose *et al.*, 2018 ▸; Assalauova *et al.*, 2020 ▸). An important step in this data processing pipeline is single-hit classification. Only diffraction patterns that contain the scattering signal of a single particle are of interest for further analysis. In our previous work (Assalauova *et al.*, 2020 ▸), this step was addressed with the expectation-maximization (EM) algorithm, first developed in cryogenic electron microscopy (Dempster *et al.*, 1977 ▸). The EM algorithm allows for unsupervised clustering of data when neither initial data assignments to clusters nor cluster parameters are known. In the end, the clusters that correspond to single hits of an investigated particle are selected manually by an expert.

The step of single-hit classification may be significantly improved by application of machine learning approaches. In recent work (Cruz-Chú *et al.*, 2021 ▸), supervised machine learning was used to map patterns into a low-dimensional manifold representation in which the authors were able to separate single from non-single hits through transformation into a bimodal distribution. In the computer vision domain, convolutional neural networks (CNNs) have become the *de facto* state of the art in image classification (Krizhevsky *et al.*, 2012 ▸), object detection (Szegedy *et al.*, 2013 ▸) and image segmentation (Long *et al.*, 2015 ▸). Thus, it is unsurprising that CNN-based solutions have been recently successfully applied in our domain: specifically, the classification of diffraction patterns in tomography experiments at synchrotron sources (Yang *et al.*, 2020 ▸) and in coherent diffraction imaging experiments at synchrotron facilities (Wu, Yoo *et al.*, 2021 ▸; Wu, Juhas *et al.*, 2021 ▸) and at XFELs (Shi *et al.*, 2019 ▸; Zimmermann *et al.*, 2019 ▸). As we showed in our previous work (Ignatenko *et al.*, 2021 ▸), a CNN-based solution can be successfully applied to the single-hit diffraction pattern classification step (Fig. 1 ▸, blue arrows).

In this work, we further develop this approach (Fig. 1 ▸, red arrows). By classifying single hits first, computationally intensive steps of the pipeline, such as size filtering and EM-based selection, need only be performed on a fraction of the initially collected patterns, saving substantial computational resources. In addition, the proposed scheme allows the classification of newly collected patterns independently, without the need to recompute from the beginning (as would be required by pure EM-based selection). This is particularly useful as experimentalists have the possibility to plan the experiment as it goes and stop it whenever a sufficient number of single hits have been collected, thereby saving precious beamtime at the XFEL facility.

## 2. SPI experiments and data analysis

**Figure 2:**
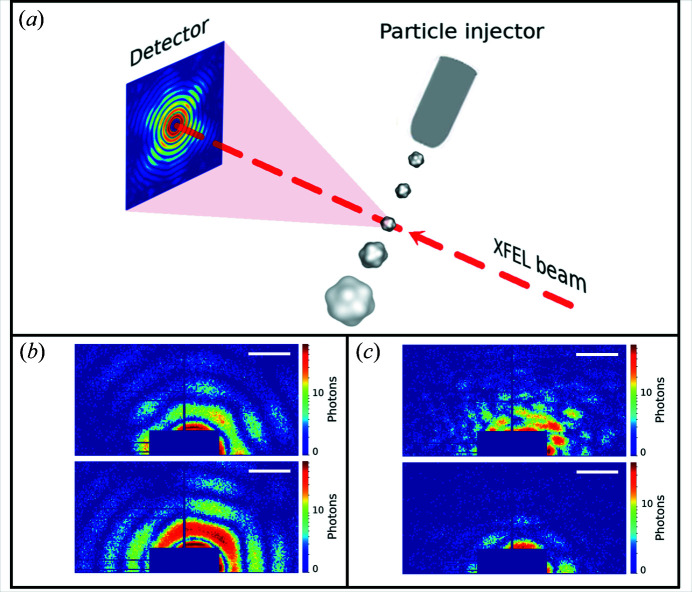
(*a*) Schematic representation of an SPI experiment. The incoming XFEL beam interacts with the virus injected by the particle injector. The particle is destroyed afterwards owing to Coulomb explosion. X-ray radiation from the non-destroyed virus is scattered to the detector positioned in the far field. (*b*) Examples of single hits. (*c*) Examples of non-single hits. The diffraction patterns in (*b*) and (*c*) are shown in logarithmic scale; an area of size 192 × 96 pixels from the whole diffraction pattern is shown. The scale bars in (*b*) and (*c*) correspond to 0.2 nm^−1^.

The SPI experiment [Fig. 2 ▸(*a*)] was performed at the Atomic Molecular Optics instrument (Ferguson *et al.*, 2015 ▸; Osipov *et al.*, 2018 ▸) at the LCLS in the framework of the SPI initiative (Aquila *et al.*, 2015 ▸). Samples of PR772 bacteriophage (Reddy *et al.*, 2017 ▸; Li *et al.*, 2020 ▸) were aerosolized using a gas dynamic virtual nozzle in a helium environment (Nazari *et al.*, 2020 ▸). The particles were injected into the sample chamber using an aerodynamic lens injector (Hantke *et al.*, 2014 ▸; Benner *et al.*, 2008 ▸). The particle stream intersected the pulsed and focused XFEL beam. The XFEL had a repetition rate of 120 Hz, an average pulse energy of ∼2 mJ, a focus size of ∼1.5 µm and a photon energy of 1.7 keV (wavelength 0.729 nm). Diffraction patterns were recorded by a pn-type CCD detector (Strüder *et al.*, 2010 ▸) mounted at 0.130 m distance from the interaction region. The detector consisted of two panels. The size of each panel was 512 by 1024 pixels with a pixel size of 75 × 75 µm. The scattering signal was only recorded by one (upper) of the two detector panels (the lower one was not operational during the experiment owing to an electronic fault).

The total number of diffraction patterns collected during the experiment was 1.2 × 10^7^ (data set *D*_0_ in Table 1 ▸) (Li *et al.*, 2020 ▸). Out of those images, only a small fraction contained any scattering patterns. To isolate such patterns, hit finding was performed using the software *psocake* in the *psana* framework (Damiani *et al.*, 2016 ▸). As a result, 191 183 diffraction patterns (data set *D* in Table 1 ▸) were selected as hits from the initial set of experimental data (Li *et al.*, 2020 ▸). Manual selection of single-hit diffraction patterns was performed on the data set *D* (data set *D*_M_ in Table 1 ▸), which resulted in 1393 single-hit diffraction patterns [see Li *et al.* (2020 ▸)]. This selection was used as a ground truth for training and evaluating the CNN in this work. In our previous work (Assalauova *et al.*, 2020 ▸), we used the EM-classification step (see Fig. 1 ▸, black arrows) to select single-hit diffraction patterns, which gave us the *D*_EM_ selection (see Table 1 ▸).

## 3. Methods

### 3.1. CNN description

A CNN consists of a succession of convolutional layers, interlaced with nonlinearities. Like most supervised machine learning models, CNNs need to be trained using a set of annotated data stemming from the task that they are intended to solve. As part of the training process, the parameters of the CNN will be tuned to enable it to learn the requested task. Here, the vast majority of parameters are represented by the weights of the convolutional kernels. Training takes place via stochastic gradient descent, where images from the training set are given to the network (forward pass) and the output of the network is compared with the reference annotation through a loss function. Then, the gradients of that loss function with respect to each of the model’s parameters are computed (backwards pass) and used to update the weights. This process is repeated many times until the model converges, *i.e.* the training loss no longer decreases. The advantage of CNNs over traditional image analysis methods is that the experimenter no longer needs to manually define and compute informative feature representations of the input. This is handled intrinsically by the convolutional layers and learned automatically as part of the training process. As a consequence, CNNs have far greater capabilities in terms of the complexity of tasks they can solve but often require a larger number of annotated example images.

### 3.2. CNN architecture

**Figure 3:**
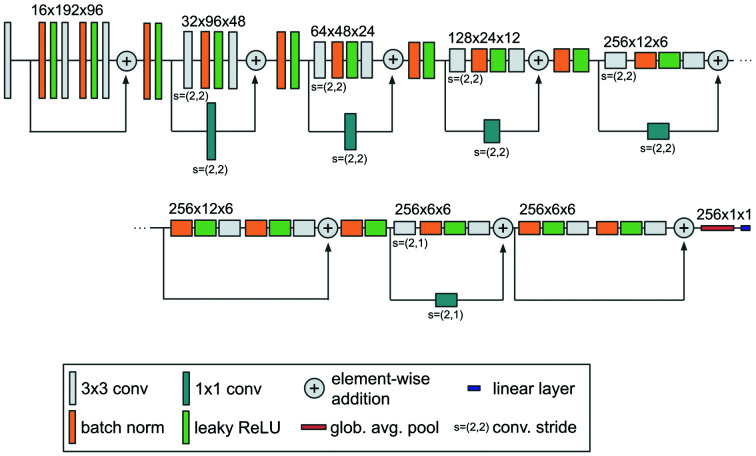
Network architecture. We use a pre-activation ResNet-inspired architecture. It takes patches of size 192 × 96 as input and processes them in a sequence of eight pre-activation residual blocks. Downsampling is implemented via strided convolution. The architecture is initialized with 16 filters and doubles the number of filters with each downsampling operation up to a maximum of 256. Global average pooling reduces the final feature representation (shape 6 × 6) to a vector that is then used by the classification layer to distinguish single from non-single hits. The size of the feature representations is indicated above each residual block. 16 × 192 × 96 here denotes 16 convolutional filters with a feature representation of size 192 × 96.

The network architecture used in this work is shown in Fig. 3 ▸. It is inspired by the pre-activation ResNet-18 (He *et al.*, 2016 ▸) and was selected on the basis of initial experiments on the training data set. The network processes patches of size 192 × 96 and is initialized with 16 convolutional filters. The number of filters is doubled with each downsampling up to a maximum of 256. Downsampling is implemented as strided convolution. We use leaky ReLU activation functions (Xu *et al.*, 2015 ▸) and standard batch normalization (Ioffe & Szegedy, 2015 ▸). The final feature map has a size of 6 × 6, which is aggregated through global average pooling into a vector that is then processed by a linear layer to distinguish single and non-single hits.

### 3.3. CNN evaluation metrics

As evaluation metrics we used precision, recall and the F1 score. These values are defined through true positive (TP), false positive (FP) and false negative (FN) predictions. The definition of the evaluation metrics is as follows:

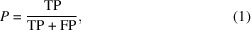




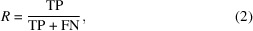

where* P *is the precision and *R* is the recall metrics. The F1 score is the harmonic mean of the precision and recall:

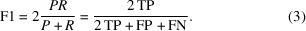

Owing to the pronounced class imbalance in our data set (a small number of single hits in comparison with a large number of non-single hits), we mainly use the F1 score for evaluating our models. In addition, we report the number of single hits.

### 3.4. Training, validation and test procedure in CNN classification

We use a training data set that is representative of the modified workflow introduced in Section 1, where the experimentalist identifies a limited number of single hits at the beginning of the experiment. Taking into account the annotation effort that would be required, we chose to use 100 single hits and a number of non-single hits that corresponds to the number of images the experimentalist would have seen until the required number of single hits was collected (see Table 1 ▸). In accordance with the class ratio of the data set used here (approximately 1:200), our training set (*D*_tr_) consists of 100 single and 19 900 non-single hits. All hits were sampled randomly without replacement. We used the manual selection *D*_M_ as a ground truth.

To prepare our data for the CNN, all diffraction patterns were cropped to an area of size 192 × 96 pixels [see supporting information Fig. S1, and Figs. 2 ▸(*b*) and 2 ▸(*c*). All images were normalized by subtraction of the training-data-set (20 000 data) mean value (μ = 0.342) and divided by the standard deviation of the same data set (σ = 2.336).

During method development, our models were trained and validated through stratified fivefold cross-validation on the set of 20 000 training examples. We report final results on the test set (*D*_test_) consisting of the 171 183 remaining patterns (1293 single and 169 890 non-single hits) (see supporting information Section S3.3)

We trained the network with stochastic gradient descent using the Adam optimizer (Kingma & Ba, 2014 ▸), a minibatch size of 64 and an initial learning rate of 10^−4^. The standard cross-entropy loss function was used. Samples within minibatches were sampled randomly with replacement. We modified the sampling probabilities such that on average 2% of the presented samples are single hits. We defined an epoch as 50 training iterations and trained for a total of 1000 epochs (50 000 iterations). The learning rate was reduced each epoch according to the polynomial-learning-rate schedule presented by Chen *et al.* (2018 ▸) (see also supporting information S3.1).

#### 3.4.1. Data augmentation

Owing to the limited number of training cases, extensive data augmentation is performed on the fly during training using the *batchgenerators* framework (Isensee *et al.*, 2020 ▸). Specifically, we used random rotations, scaling, elastic deformation, gamma augmentation, Gaussian noise, Gaussian blur, mirroring, random shift and cutout (DeVries & Taylor, 2017 ▸) (for details regarding the data augmentation pipeline, see supporting information Section S3.4).

#### 3.4.2. Inference

For model development we used stratified fivefold cross-validation on the training set. The resulting five models are used as an ensemble for test set predictions. We further use test-time data augmentation (mirroring). Ensembling is implemented via softmax averaging, followed by thresholding at 0.5 to obtain the final predictions (see supporting information Sections S3.2 and S3.3).

### 3.5. CNN variant: identifying more single hits

The CNN model described above is optimized for maximizing the F1 score on our training cross-validation. We subsequently refer to it as ‘MaxF1’. In addition, we trained a second CNN model that predicts a larger number of single hits (‘moreSH’) and leans more towards higher recall values. To achieve that, we made modifications to the sampling strategy as well as the loss function. Specifically, we increased the probability of selecting single hits when constructing the minibatches from 2 to 5% and made use of a weighted cross-entropy loss which weights samples of ground-truth single hits higher during loss computation (weights 0.1 and 0.9 for non-single hits and single hits, respectively). For both models (MaxF1 and moreSH), we used the same augmentation and inference scheme.

### 3.6. Comparison metrics of different data selections

To compare different data selections, we also looked at the intersection over union α metric, which can be described as

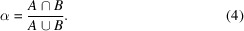

Here *A* and *B* are two sets of data, and signs 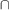 and 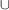 mean intersection and union of these two data sets.

As a result of single-hit classification, we obtained data selections with different numbers of diffraction patterns. In order to compare these selections, we plotted and analysed the power spectral density (PSD) function, *i.e.* the angular averaged intensity. To quantify the contrast values of the PSD functions for each selection, we introduced the following metric, which describes the mean difference between the local minima and maxima over the first three pairs:

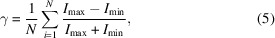

where *N* = 3 is the number of pairs, and *I*_max_ and *I*_min_ are values of the PSD function for the maxima and minima, respectively. By looking at the PSD functions and the corresponding contrast values we can compare various single-hit selections and analyse which one has more features.

### 3.7. Particle size determination

Particle size filtering is also an important part of the SPI data analysis workflow (see Fig. 1 ▸ and supporting information Section S4). It can help to remove unnecessary diffraction patterns corresponding to other particles apart from the viruses under investigation. In the previous approach (Fig. 1 ▸, black arrows), particle size determination was carried out on the entire data set *D* prior to applying the EM classification, and thus the single-hit classification was performed only on particle sizes between 55 and 84 nm [see Assalauova *et al.* (2020 ▸)]. In this work we used the CNN classification after the initial preprocessing step and particle size filtering was applied afterwards. Here we used the same results for the virus size estimation as Assalauova *et al.* (2020 ▸), and the same virus size range (55–84 nm) was considered here.

## 4. Results

### 4.1. CNN performance

Table 2 ▸ summarizes the performance of our CNNs on the training set cross-validation. The MaxF1 configuration obtains balanced precision and recall and an F1 score of 0.645. The number of predicted single hits (120) is close to the number of single hits (100) in this data set. The moreSH configuration, however, trades a higher recall with lower precision, resulting in an overall decreased F1 score of 0.536. As expected, the number of predicted single hits is higher, being 221 in this case.

Test set predictions (see Table 3 ▸) were obtained by ensembling the five models obtained during cross-validation (see supporting information Sections S3.2 and S3.3). On the test set (171 183 patterns), the MaxF1 configuration obtained an F1 score of 0.731 with balanced precision and recall. Interestingly, the F1 score is substantially higher than that on the training set cross-validation, which we attribute to the use of ensembling. The predicted number of single hits (1257 patterns) is close to the number of single hits (1393 patterns) in the reference set *D*_M_.

The moreSH configuration, as expected, again displays an imbalance between precision and recall. Overall, its recall is higher (0.841 versus 0.721), but its F1 score is lower at 0.644 (versus 0.731). Again, as expected, the number of predicted single hits is larger (2086 patterns).

On a workstation equipped with an AMD Ryzen 5800X CPU, 32 GB of RAM and an Nvidia RTX 3090 GPU, training each individual model took less than 25 min (<2.5 h for all five models in the cross-validation). The inference speed was ∼450 diffraction patterns per second for the ensemble and with test-time data augmentation (five models and mirroring along all axes for a total of 20 predictions per pattern). Predicting the 171 183 test patterns took less than 7 min. If faster inference is required, single-model prediction without test-time augmentation can be used to increase the throughput to ∼8700 patterns per second. Training required merely 3.5 GB of VRAM, and a much smaller GPU than the RTX3090 used here would have been sufficient as well.

### 4.2. PSD comparison, EM and particle size filtering

**Figure 4:**
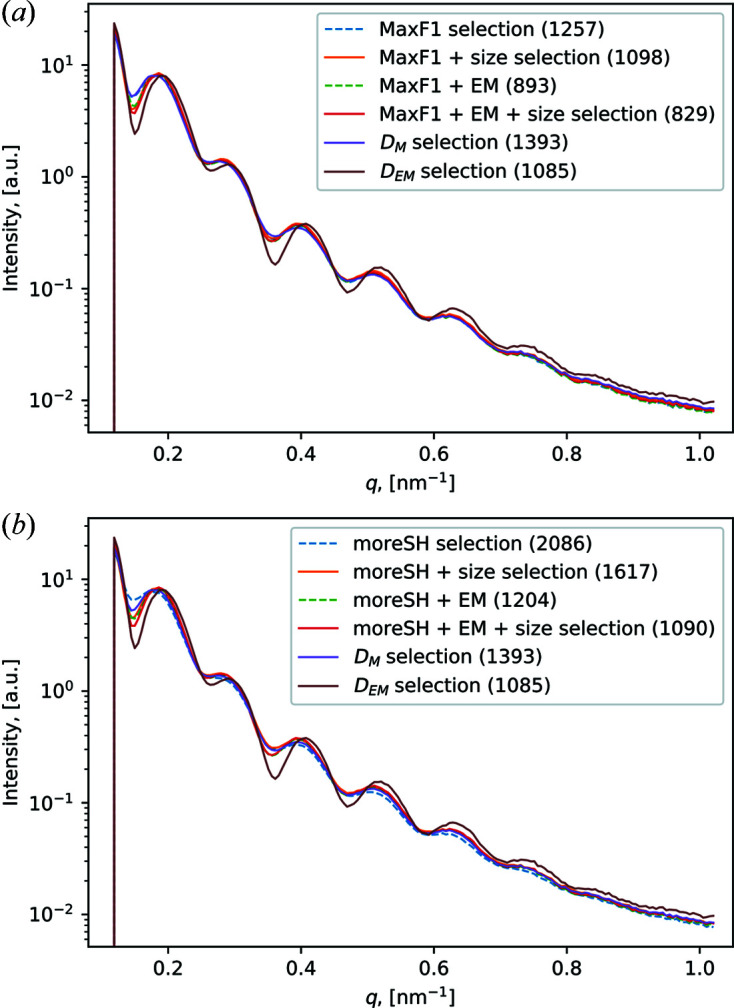
PSD functions for the different data sets. (*a*) PSD functions for the MaxF1 data selection. (*b*) PSD functions for the moreSH data selection. Blue dashed line – the whole selection, orange line – selection with size filtering applied, green dashed line – selection with the EM algorithm applied, red line – selection with the EM algorithm and size filtering applied. Both panels (*a*) and (*b*) contain the PSD functions of the *D*_M_ (puple line) and *D*_EM_ (brown line) selections. In the legend, the number of diffraction patterns for each selection is shown in brackets.

As a result of CNN classification, we obtained two data sets: MaxF1 and moreSH with the number of single-hit diffraction patterns 1257 and 2086, respectively (see Table 4 ▸). Plotted PSD functions for both selections are shown in Fig. 4 ▸ (blue dashed lines). Additionally, we plotted the PSD functions for the *D*_M_ and *D*_EM_ selections (Assalauova *et al.*, 2020 ▸), containing 1393 and 1085 diffraction patterns, respectively (Fig. 4 ▸, purple and brown solid lines). The corresponding number of diffraction patterns and PSD contrast values for all four data sets (MaxF1, moreSH, *D*_M_ and *D*_EM_ selection) are given in Table 4 ▸. From Fig. 4 ▸ we observe the same number of fringes as in our previous paper. However, the contrast values were lower in the case of CNN classification in comparison with EM classification. As expected, the PSD functions for MaxF1 and moreSH mimic the behaviour of the PSD function of the *D*_M_ selection which was used as the ground truth for CNN training.

In order to increase the PSD contrast of the CNN selection, we applied EM-based selection to the MaxF1 and moreSH data sets (see supporting information Section S5). The results of this additional selection are summarized in Fig. 4 ▸ (green dashed lines) and Table 4 ▸ with notation ‘+ EM’. The contrast for moreSH + EM selection showed a substantial improvement (0.64 versus 0.59 without EM), and we also observed a slight improvement for the MaxF1 + EM selection (0.64 versus 0.63 without EM). At the same time, the EM selection (Assalauova *et al.*, 2020 ▸) still has the best result in terms of contrast.

**Figure 5:**
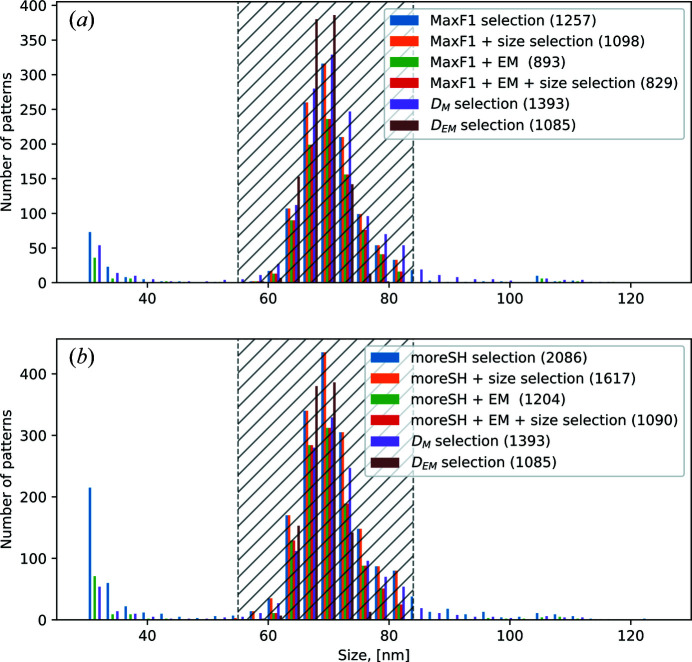
Particle size histograms for different data sets. (*a*) Particle size histogram for the MaxF1 data selection. (*b*) Particle size histogram for the moreSH data selection. Blue bins – the whole selection, orange bins – selection with size filtering applied, green bins – selection with the EM algorithm applied, red bins – selection with the EM algorithm and size filtering applied. In both panels (*a*) and (*b*), the dashed areas indicate the particle size range from 55 to 84 nm; the *D*_M_ selection is shown in purple bins; the *D*_EM_ selection is shown in brown bins. In the legend, the number of diffraction patterns for each selection is given in brackets.

The EM classification carried out by Assalauova *et al.* (2020 ▸) was performed on a size range of viruses from 55 to 84 nm, which was determined prior to EM classification. To perform particle size analysis in this work, we first plotted histograms of the particle size distribution for each data set (MaxF1 with/without EM algorithm applied, moreSH with/without EM algorithm applied) in Fig. 5 ▸. Each data selection consists of diffraction patterns within a wide size range. This means that, even after single-hit classification (with/without EM algorithm), the data sets contain diffraction patterns that correspond to particles of different sizes. To be consistent with our previous work, the size range from 55 to 84 nm was considered for further analysis and particle size selection was applied. The corresponding PSD functions are plotted in Fig. 4 ▸ (solid orange and red lines), and the resulting numbers of diffraction patterns and contrast values are summarized in Table 4 ▸ with notation ‘+ size selection’.

Fig. 4 ▸(*a*) and Table 4 ▸ show that for the MaxF1 data set the particle size filtering did not change the contrast values (= 0.64). However, for the selection moreSH with the EM algorithm applied the particle size filtering gave the best PSD contrast value (= 0.65).

Even though we were able to increase the PSD contrast through different classification strategies and particle size filtering, we, unfortunately, reduced the number of diffraction patterns along the way. For the MaxF1 data set we started from a data set of 1257 patterns and finally came to 827 patterns. For the moreSH selection, we started with 2086 patterns and finally came to 1090 patterns. In the context of our data processing pipeline, where a large number of single hits is required to get reliable results, this can be detrimental.

In the following, we will consider four final data sets: MaxF1 with size filtering applied [Fig. 4 ▸(*a*), orange solid line; Fig. 5 ▸(*a*), orange histogram], MaxF1 with the EM algorithm and size filtering applied [Fig. 4 ▸(*a*), red solid line; Fig. 5 ▸(*a*), red histogram], moreSH with size filtering applied [Fig. 4 ▸(*b*), orange solid line; Fig. 5 ▸(*b*), orange histogram], and moreSH with the EM algorithm and size filtering applied [Fig. 4 ▸(*b*), red solid line; Fig. 5 ▸(*b*), red histogram].

### 4.3. Intersection over union comparison

We also compared diffraction patterns in our four final data sets in terms of the intersection over union metric. The values obtained for different pairs of data sets are shown in Table 5 ▸. In addition, we calculated the intersection over union over three selections – MaxF1 with size filtering applied, moreSH with size filtering applied and *D*_EM_ selection – which gave the intersection over union α = 29% with 575 diffraction patterns in the intersection. Another three selections – MaxF1 with EM algorithm and size filtering applied, moreSH with the EM algorithm and size filtering applied, and *D*_EM_ selection – gave the intersection over union α = 29% with 469 diffraction patterns. We think that this choice of diffraction patterns in the intersection of three data selections is providing us with the most important diffraction patterns that contain the features of virus structure from all data selections.

### 4.4. Orientation determination

The next step of the workflow for SPI analysis after single-hit classification is orientation determination of the diffraction patterns (see Fig. 1 ▸). In SPI experiments particles are injected into the X-ray beam in random orientations, so to retrieve a 3D intensity map of the virus from the selected 2D diffraction patterns, orientation recovery has to be done. The expand–maximize–compress algorithm (Loh & Elser, 2009 ▸) in the software *Dragonfly* (Ayyer *et al.*, 2016 ▸) was used to retrieve the orientation of each diffraction pattern and to combine them into one 3D intensity distribution of the PR772 virus. We retrieved the orientation of all previously selected data sets with the size filtering applied, with and without the EM classification.

**Figure 6:**
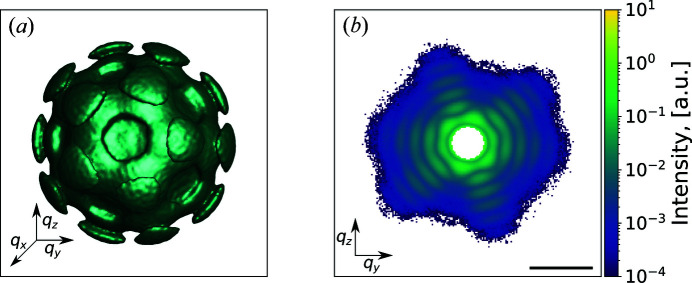
Reciprocal space representation for the MaxF1 selection with the EM algorithm and size filtering applied. (*a*) 3D intensity distribution of the virus in reciprocal space after background subtraction. (*b*) 2D cut of the distribution. All diffraction patterns are shown in logarithmic scale. The black scale bar denotes 0.5 nm^−1^.

Visual inspection does not allow us to see a significant difference between data sets (MaxF1 and moreSH with/without the EM algorithm applied, and with size filtering applied). However, for all four data sets the background at high *q* values is clearly seen (see supporting information Fig. S4). Background subtraction is a common task in SPI data analysis and several techniques have already been developed (Rose *et al.*, 2018 ▸; Lundholm *et al.*, 2018 ▸; Ayyer *et al.*, 2019 ▸). In this work we defined the level of the background as the mean signal in the high-*q* region, where the presence of meaningful signal from the particle is negligible. The orientation determination results after background subtraction on the MaxF1 CNN selection with the EM and size filtering applied is shown in Fig. 6 ▸ (for other data sets see supporting information Fig. S5).

### 4.5. Phase retrieval and reconstructions

The next and the final step in our workflow is phase retrieval and reconstruction of the electron density of our virus particle from the 3D reciprocal space data (see Fig. 1 ▸). Since the experimental measurements provide only the amplitude of the complex-valued scattered wavefield, we applied iterative phase retrieval algorithms (Fienup, 1982 ▸; Marchesini, 2007 ▸) in order to determine the 3D structure of the virus particle. The following algorithms were used in this work for the phase retrieval: continuous hybrid input–output (Fienup, 2013 ▸), error reduction (Fienup, 1982 ▸), Richardson–Lucy deconvolution (Clark *et al.*, 2012 ▸) and shrink-wrap (Marchesini *et al.*, 2003 ▸).

We proceeded in the same way as Assalauova *et al.*( 2020 ▸). The phase retrieval procedure consisted of two steps. In the first step, the central gap in the 3D intensity map of the virus that originated from the masking of the initial 2D diffraction patterns was filled. Running 3D reconstruction with a freely evolving central part produced a signal in the masked region which was used further. In the second step, the 3D intensity maps with the filled central part were used to perform phase retrieval. We first performed 50 reconstructions for each intensity map and then used mode decomposition (Khubbutdinov *et al.*, 2019 ▸; Assalauova *et al.*, 2020 ▸) to determine the final 3D electron density structure of the virus.

**Figure 7:**
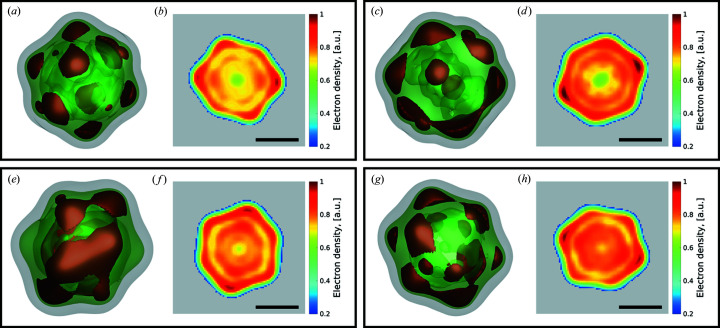
PR772 virus reconstructed from the different data sets. (*a*)–(*d*) Reconstruction of single-hit diffraction patterns selected by MaxF1 with size filtering applied (*a*), (*b*) and MaxF1 with the EM algorithm and size filtering applied (*c*), (*d*). (*e*)–(*h*) Reconstruction of the single-hit diffraction patterns selected by moreSH with size filtering applied (*e*), (*f*) and moreSH with the EM algorithm and size filtering applied (*g*), (*h*). (*a*), (*c*) The 3D inner structure of the virus with 88% (brown), 75% (green) and 20% (grey) levels of intensity for the MaxF1 selections. (*e*), (*g*) The 3D inner structure of the virus with 86% (brown), 75% (green) and 20% (grey) levels of intensity for the moreSH selections. (*b*), (*d*), (*f*), (*h*) 2D slices of the corresponding structure with the same scale bar of 30 nm. For visual representation, each virus structure was upsampled three times.

The final virus structure for each data selection, obtained in the described way, is shown in Fig. 7 ▸. All expected features are present in these reconstructions: the icosahedral structure of the virus, higher density in the capsid part of the virus and reduced density in the central part. The resolution of the obtained images, evaluated by the Fourier-shell correlation (FSC) method, gave values from 6 to 8 nm (see supporting information Section S7). The slightly higher resolution determined in this work relative to our previous work (6.9 nm) may be related to the comparatively small number of diffraction patterns used in the FSC method. As we observe in Figs. 7 ▸(*a*)–7 ▸(*d*), the electron densities of the virus in the CNN MaxF1 selection with size filtering and MaxF1 selection with EM selection plus size filtering are practically identical. We see small differences from the previous electron density in the CNN moreSH selection with size filtering and moreSH with EM selection plus size filtering [Fig. 7 ▸(*e*)–7 ▸(*h*)]. At the same time, the central slice in all four reconstructions [Figs. 7 ▸(*b*), 7 ▸(*d*), 7 ▸(*f*) and 7 ▸(*h*)] is practically the same, the capsid layer being the same size. Since we have 400–500 diffraction patterns in common with the considered data selections and our previous work (Assalauova *et al.*, 2020 ▸), we can assume that these were the ones that contributed to and shaped the final reconstructed results in such a common way for all five data selections.

## 5. Discussion and summary

Our studies with the CNN-based single-hit classification implemented within the SPI data analysis workflow resulted in a reasonable structure reconstruction of the virus PR772 (see Fig. 7 ▸).

We compared two competing CNN selections, MaxF1 and moreSH. The MaxF1 selection was intended to select single hits with an optimal F1 score. The selection moreSH was optimized for finding more single-hit diffraction patterns (high recall). Both selections were refined by applying the EM algorithm and limiting the selection to particle sizes in the range 55–84 nm (Table 4 ▸). Driven by the need for many single hits in the reconstruction pipeline, the moreSH configuration was conceived with the intention of missing as few single hits as possible; the selection was cleaned up afterwards using EM selection and size filtering, in the hope of achieving a higher resolution than could be obtained with the MaxF1 counterpart. Unfortunately, this goal was missed: MaxF1 yielded approximately the same resolution even though the moreSH approach resulted in 1090 selected single hits instead of the 829 found by MaxF1 (with EM and size selection applied). We therefore conclude that optimizing balanced precision and recall through maximizing the F1 score is a suitable target for model development.

CNNs learn from their given training data set. Unfortunately, the selection provided by Li *et al.* (2020 ▸) which was used for this purpose here, as any other manual selection, may be subjective. In addition, the task of identifying single hits is not necessarily identical to the task of finding the ideal set of patterns needed for reconstruction. In an ideal world, the CNNs should be trained with the patterns ideally suited for reconstruction. Until we identify a way of obtaining ideal patterns from a subset of our data, subjectively selected single hits are the next-best solution.

The particle size filtering step is quite important and has to be applied throughout the SPI analysis pipeline. A real experiment might run in the following way. A trained person will select a number of single hits and non-single hits and then will run the CNN selection on the diffraction patterns coming from the experimental stream. After size filtering, this selection will be uploaded to the SPI workflow as shown in Fig. 1 ▸, and the electron density of a single particle will be obtained as a result.

Reconstructing the 3D structure from a selection of single hits is expensive: both computationally and in terms of manual labour. We introduced the PSD contrast in the hope that it would constitute a good substitute measure for the quality of a selection. If successful, this would have allowed us to optimize our CNNs more directly towards identifying an optimal set of single hits for reconstruction through maximizing their PSD contrast. Comparing the PSD contrast between CNN selections, *D*_M_ and *D*_EM_ (Assalauova *et al.*, 2020 ▸) revealed that the contrast in the CNN and *D*_M_ selections is always lower than that in the *D*_EM_ selection. We initially thought that this may be problematic for the reconstructions. However, as the results in Fig. 7 ▸ demonstrate, this is not the case and our CNN selection (which mimics *D*_M_) is working well, resulting in an electron density of the PR772 virus that is similar to that obtained in our previous work (Assalauova *et al.*, 2020 ▸). These results indicate that the PSD contrast may not be a good substitute for reconstruction fidelity. Deviations from a circular shape, as are present in PR772, might explain this observation.

We have proposed an SPI workflow that uses a CNN-based single-hit classification at an early stage of the data analysis pipeline. This approach can be beneficial not only because it can be run during SPI experiments but also because it can significantly reduce the number of diffraction patterns for further processing. That is important for data storage, as the size of collected data sets during one experiment at a megahertz XFEL facility can easily reach several petabytes. Another convenience of using CNNs for single-hit classification is that the network can be trained on a relatively small quantity of data at the beginning of the SPI experiment and can be simply applied throughout the rest of the experiment.

Introducing non-standard AI-based solutions into an established SPI analysis workflow may be beneficial for the future development of SPI experiments. Here we have demonstrated the use of CNNs at the single-hit diffraction-pattern classification step, which can be applied not only after the experiment but, importantly, also during the experiment and can significantly reduce the size of data storage for further analysis stages. That could be an important advantage with the development of high-repetition-rate XFELs (Decking *et al.*, 2020 ▸) with data collection with the megahertz rate (Sobolev *et al.*, 2020 ▸). Handling experimental data with CNNs also saves computational time: once the CNN is trained and new data are obtained, there is no need to retrain the CNN again as is needed with other classification approaches.

## 6. Data and code availability

The experimental data sets used in this publication are publicly available: https://www.cxidb.org/id-156.html. They were preprocessed (background correction, center estimation) as described by Bobkov *et al.* (2020 ▸) using the code available at https://gitlab.com/spi_xfel (see spi_processing section). For convenience, the preprocessed data are also available at https://zenodo.org/record/6451444 (Assalauova *et al.*, 2022 ▸).

The code for training the CNN and running predictions on our test set is available at https://gitlab.hzdr.de/hi-dkfz/applied-computer-vision-lab/collaborations/desy_2021_singleparticleimaging_cnn.

## 7. Related literature

The following additional literature is cited in the supporting information: Harauz & van Heel (1986 ▸); van Heel & Schatz (2005 ▸); Scheres *et al.* (2005 ▸).

## Supplementary Material

Supporting information file. DOI: 10.1107/S1600576722002667/te5090sup1.pdf

Experimental data sets used in this publication: https://doi.org/10.11577/1645124

Preprocessed data (background correction, center estimation): https://doi.org/10.5281/zenodo.6451444

## Figures and Tables

**Table:** 

Data set	No. of diffraction patterns	
Initial data set, *D*_0_	1.2 × 10^7^	
Hit finding procedure, *D*	191 183	
Manual selection of single hits, *D*_M_	1393	
Selection by EM algorithm, *D*_EM_	1085	

**Table:** 

	Single hits	Non-single hits
Training and validation data set, *D*_tr_	100	19 900
Test data set, *D*_test_	1293	169 890

**Table 2:** Five-fold cross-validation results (*N* = 20 000 training samples)

	MaxF1	moreSH
F1 score	0.645 ± 0.074	0.536 ± 0.018
*P* (precision)	0.591 ± 0.062	0.391 ± 0.023
*R* (recall)	0.710 ± 0.096	0.860 ± 0.065
Predicted single hits	120	221

**Table 3:** Test set results (*N* = 171 183 test samples)

	MaxF1	moreSH
F1 score	0.731	0.644
*P* (precision)	0.741	0.522
*R* (recall)	0.721	0.841
Predicted single hits	1257	2086

**Table 4:** Number of diffraction patterns in different data sets of single hits and PSD contrast values for each of them

Data set	No. of diffraction patterns	PSD contrast
MaxF1	1257	0.63
MaxF1 + EM	893	0.64
MaxF1 + size selection	1098	0.64
MaxF1 + EM + size selection	829	0.64
moreSH	2086	0.59
moreSH + EM	1204	0.64
moreSH + size selection	1617	0.62
moreSH + EM + size selection	1090	0.65
*D*_M_	1393	0.59
*D*_EM_	1085	0.71

**Table 5:** Number of diffraction patterns in intersections of different pairs of data sets The initial number of diffraction patterns in the sets is shown in brackets. In the second line, the intersection over union α is shown.

	MaxF1 + size selection (1098)	MaxF1 + EM + size selection (829)	moreSH + size selection (1617)	moreSH + EM + size selection (1090)	*D*_M_ (1393)	*D*_EM_ (1085)
MaxF1 + size selection (1098)	1098	829	1097	878	875	575
	100%	75%	68%	67%	54%	36%

MaxF1 + EM + size selection (829)	829	829	829	730	678	485
	75%	100%	51%	61%	44%	34%

moreSH + size selection (1617)	1097	829	1617	1090	1006	686
	68%	51%	100%	67%	50%	34%

moreSH + EM + size selection (1090)	878	730	1090	1090	791	651
	67%	61%	67%	100%	47%	43%

*D*_M_ (1393)	875	678	1006	791	1393	574
	54%	44%	50%	47%	100%	30%

*D*_EM_ (1085)	575	485	686	651	574	1085
	36%	34%	34%	43%	30%	100%
