# Supplementary material for: Classification of diffraction patterns using a convolutional neural network in single-particle-imaging experiments performed at X-ray free-electron lasers
Source: J Appl Crystallogr. 2022 Apr 22;55(Pt 3):444–54. doi: 10.1107/S1600576722002667 (PMC9172041; doi:10.1107/S1600576722002667)
Supplement: Supplementary file 1 [file j-55-00444-sup1.pdf]

JOURNAL OF APPLIED CRYSTALLOGRAPHY

Volume 55 (2022)

Supporting information for article:

Classification of diffraction patterns using a convolutional neural network in single-particle-imaging experiments performed at X-ray free-electron lasers

Dameli Assalauova, Alexandr Ignatenko, Fabian Isensee, Darya Trofimova and Ivan Vartanyants

Supporting information

S1. Data cropping

Data was prepared before applying CNN-based single hit diffraction patterns classification. The region of interest on all diffraction patterns from the data set D was cropped as it is shown in Fig. S1.

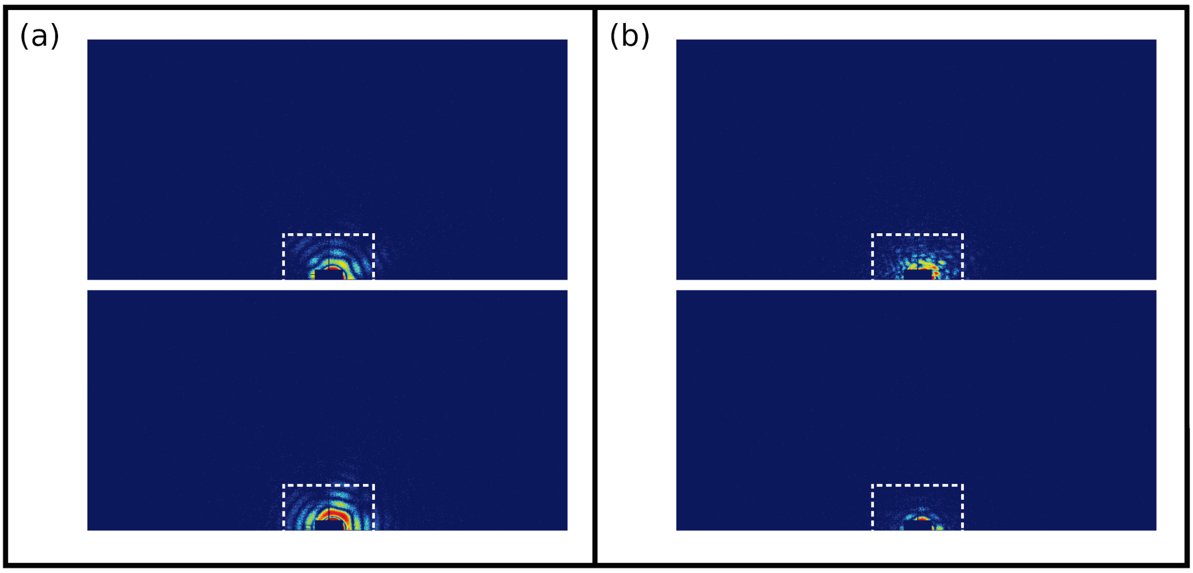

Figure S1 Illustration of data cropping before sending to the input of a CNN. The center of diffraction pattern is located around the center of the bottom part of the only operational detector plane with dimensions in pixels 1024 × 512. The area surrounding the center of each diffraction pattern with dimensions in pixels 192 × 96 is cropped (white dotted rectangle). The cropped part is used as an input of a CNN. (a) Single hit examples, (b) non-single hit examples.

S2. VGG

The main studies in the field of CNN classification of single hits were carried out with the network architecture pre-activated ResNet-18 described in the main part of the paper. In order to investigate an effect of CNN depth required for the specific task of single hit classification, a VGG-style network was implemented within the same pipeline. This network is realized as a plain sequence of convolutional layers organized in four downsampling stages (Fig. S2). The activation function is ReLU. Batch normalization layer precedes each convolutional layer, except the first one. Dimensionality reduction is realized via maximal pooling. The number of filters in the convolutional layers of the first stage is 16. It rapidly grows up to 256 at the last stage. This growth is intentionally fast. It allows to extract more higher level features while preventing the network from growing in

depth. Global average pooling is used to linearize the final feature representation of the shape 12 x 16 to a feature vector used for classification.

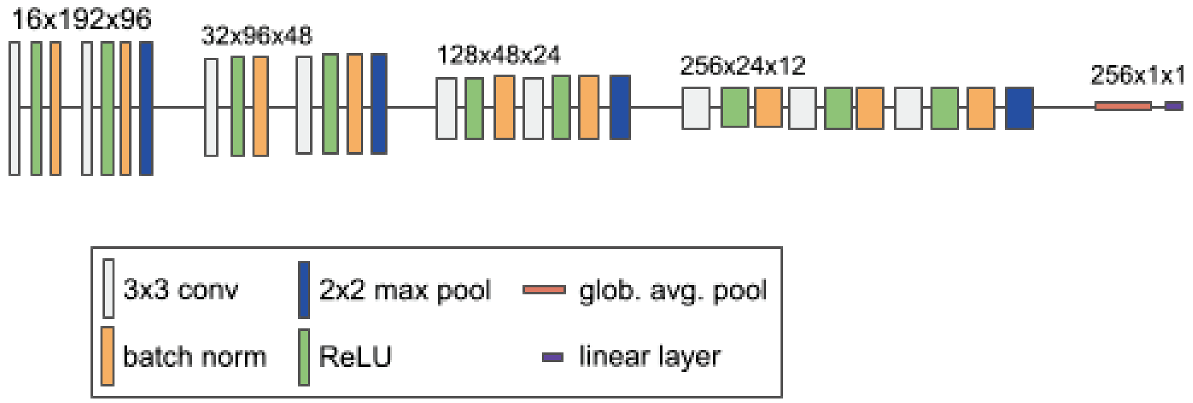

Figure S2 VGG-style network architecture. We use a simple VGG-style network for comparison. It has the same input size of 192 x 96. It processes the input in four downsampling stages. Downsampling is implemented via maximal pooling. The convolutional layer of the first stage has 16 filters. The number grows up to 256 filters for the fourth stage. Global average pooling is used to linearize the final feature representation of the shape 12 x 16 to a feature vector used for classification.

Training, validation and test follow the same procedure described for Resnet-18. The results for relevant metrics for five-fold cross-validation is shown in Table S1. Test performance metrics for the VGG-style network (Table S2) is similar to that of Resnet-18 (Table 3 in the main text). This is an indication that the choice of network depth within the investigated limit has negligible effect. Thus, a simple VGG-style network can be sufficient for the task.

Table S1 VGG five-fold cross-validation results (n=20,000 training samples).

**F1 score**	0.678
**Precision**	0.656
**Recall**	0.72
**Predicted single hits**	113

Table S2 VGG test set results (n=171,183 test samples).

**F1 score**	0.727
**Precision**	0.78
**Recall**	0.681
**Predicted single hits**	1,130

S3. Details on CNN training and validation

We train the network with stochastic gradient descent using the Adam optimizer (Kingma & Ba, 2014), a minibatch size of 64 and an initial learning rate of 10^{-4}. The standard cross-entropy loss function is used. Samples within minibatches are sampled randomly with replacement. We modify the sampling probabilities such that on average 2% of the presented samples are single hits. We define an epoch as 50 training iterations and train for a total of 1,000 epochs (50,000 iterations). The learning rate is reduced each epoch according to the polyLR schedule presented in (Chen *et al.*, 2018).

For model development we used five-fold cross-validation on the training set. The resulting five models are then used as an ensemble for test set predictions. Ensembling is implemented via softmax averaging, followed by thresholding at 0.5 to obtain the final predictions.

Below are the details on learning rate scheduler, five-fold cross-validation procedure and softmax averaging implemented to make the final prediction of the CNN model.

S3.1. Polynomial learning rate (polyLR) policy

Learning rate is one of the most important hyper-parameters in any neural network optimization process. It controls the speed of network convergence in the training process. One of the most common algorithms of minimization of loss function is stochastic gradient descent (SGD). SGD first computes the gradients of the loss function with respect to all model parameters using an algorithm called back-propagation and then updates the model weights w as follows:

$$w^{i+1} = w^i - \eta \cdot \frac{\partial L}{\partial w}, \quad \text{(S1)}$$

where L is the loss function, i is iteration number, nu is learning rate. A conventional approach to control convergence of a model is to set an initial value of learning rate and let it decrease over time. Here we use a learning rate scheduler called polynomial learning rate policy (polyLR) (Chen *et al.*, 2018). The learning rate is changed during training according to the equation:

$$\eta = \eta_0 \cdot (1 - \frac{i}{T_i})^{power}, \quad \text{(S2)}$$

where T_i is the total number of iterations during training.

S3.2. K-fold cross validation

Cross-validation is a procedure used to evaluate machine learning models on a limited dataset size, i. e. the amount of data is too small to draw robust conclusions using a conventional training and validation split. The procedure of k-fold cross-validation is the following. The entire data set available for training and validation is shuffled and split into k groups. For each unique group, the data from this group becomes the validation data set; the respective training data set consists of the other k-1 groups. As a result, there are k individual trained models. The performance metrics are then defined by average performance of these models.

We chose k=5 for developing our models. Final performance on the test set is obtained by using the resulting five models as an ensemble, as described in the following section.

S3.3. Ensembling via softmax averaging

Ensembling refers to combining predictions from multiple machine learning models. It is a commonly used strategy to reduce the variance of the models and increase the overall quality of the predictions. In the case of image classification (which is the setting used in this publication), ensembling can be implemented via softmax averaging. Here, this is implemented in the following way: Each CNN model issues a prediction for each diffraction pattern of the test data set providing single hit probability (ranging from 0 to 1). The average of five predictions, one for each model, is then the single hit probability for the ensemble. We put a threshold for the average single hit probability to obtain the final prediction. Diffraction patterns with final probability above 0.5 are classified as single hits.

S3.4. Details on data augmentation

Data augmentation is a powerful tool to improve the robustness of models trained on a limited number of training cases. By running transformations on the training cases, new images are generated that direct the models to learn better generalizing features and thus ultimately improve their generalization capabilities on the test set. We use the following transformations from the 'batchgenerators' framework[1]: random rotations, scaling, elastic deformation, gamma augmentation, Gaussian noise, Gaussian blur, mirroring, random shift and cutout.

Random rotation is a common augmentation technique when a source image is rotated clockwise or counterclockwise by some number of degrees. This changes the position of the object in the image. In

[1] https://github.com/MIC-DKFZ/batchgenerators

random rotation of the image its corners are cut off, after rotation the new corners are filled with padding.

Scaling can be done outward or inward. When scaling inward, the resultant image size is larger than the original image size. A section is cut out from the resultant image to make the size equal to the original image. When scaling outward, the size of the image is reduced, the missing part is filled with padding.

Obtaining an augmented image using elastic deformations is done in two parts. First, a random stress field is generated for horizontal and vertical directions with randomly sampled values:

$\Delta_x = G(\sigma)\cdot(\alpha \cdot Rand(n, m))$

$\Delta_y = G(\sigma)\cdot(\alpha \cdot Rand(n, m))$, (S3)

where G(σ) is the strength of the smoothing operation given by the standard deviation of the Gaussian filter σ, α is a parameter defining the maximum value for the random initial displacement, n and m are the image dimensions. After that, the stress field is applied to the image by moving each pixel to a new position (Eq. S4) using spline interpolation of order one to obtain pixel values at integer coordinates:

$$I_{deformed}(j + \Delta_x(j,k), k + \Delta_y(j,k) = I(j,k)\,, \quad \text{(S4)}$$

where I and $I_{deformed}$ are the initial and deformed images, j and k are pixel coordinates.

Gamma augmentation is a nonlinear operation used to encode and decode luminance in images, it is defined by power-law expression:

$V = AU^{\gamma}$, (S5)

where V is resultant pixel value, U is initial pixel value, A is a constant, γ is a parameter.

Gaussian noise is an additive noise type, where the intensity value in a pixel with the coordinates (x,y) for the noisy image is given by the expression:

N(x,y) = A(x,y) + B(x,y), (S6)

where the A(x,y) in the pixel value of the original image, B(x,y) is the added noise. The added value of noise is defined by probability density function of Gaussian random value is indicated in equation:

$$p(z) = \frac{1}{\sqrt{2\cdot\pi\cdot\sigma}} \cdot e^{\frac{-(z-\mu)^2}{2\cdot\sigma^2}}\,, \quad \text{(S7)}$$

where σ and μ are standard deviation and mean values, z is pixel value.

Gaussian blur is a type of image-blurring filter that uses a Gaussian function for calculating the transformation to apply to each pixel in the image. The response of the Gaussian filter in two dimensions is described by

$$g(x, y) = \frac{1}{2 \cdot \pi \cdot \sigma^2} \cdot e^{-\frac{x^2 + y^2}{2 \cdot \sigma^2}}, \qquad \text{(S8)}$$

x and y are the distances from the filter origin in the horizontal and vertical directions, respectively. σ is the standard deviation of the Gaussian distribution.

Mirroring implies flipping images along vertical and horizontal axes.

Random shift is a transformation when the image as a whole is shifted horizontally and vertically by a random number of pixels. The missing parts at the edges appeared due to these shifts being filled with padding.

Cutout is a data augmentation technique that randomly masks out square regions in images. These regions are of random size, appear in random positions in the image and filled with padding.

S4. Particle size determination

Particle size determination was implemented to the initial set *D* by fitting the power spectral density (PSD) function of each diffraction pattern with the PSD of diffraction pattern from the spherical particles in a range of sizes from 30 to 300 nm and was described in (Assalauova *et al.*, 2020).

S5. Application of EM algorithm

Task of a single hit classification in cryogenic electron microscopy (cryo-EM) is commonly solved with this approach (Dempster *et al.*, 1977; Scheres *et al.*, 2005). The EM classification algorithm is designed to distribute the whole data set into a predefined number of clusters. On each iteration, probabilities of patterns to be assigned to each cluster are calculated and cluster models are updated by weighted averaging of the associated patterns, where weights are determined by obtained probabilities. After the algorithm converges, one can manually select the required clusters which correspond to the particle under investigation.

If one considers the contrast of the PSD function as a criterion for best reconstruction, the EM algorithm outperforms CNN classification. EM-based algorithm was applied to the diffraction patterns selected by CNN: MaxF1 and moreSH data sets, containing 1,257 and 2,086 patterns respectively. Both selections were distributed into 20 classes (example of distribution for MaxF1 data set is in Fig. S3) and after 10 iterations of the algorithm, the obtained classes were inspected. Some of them clearly contained diffraction patterns of the virus and the rest ones contained other scattering. Classes of interest were selected manually by the 6-fold symmetry expected from the virus. In the case of the MaxF1 data set, classes 3, 6, 9, 12, 16, 19 (highlighted with red title) were considered to

contain patterns of interest. The final numbers of the diffraction patterns before and after applying EM-based algorithm are presented in the main text in Table 4.

Below are computing times to obtain D_{EM} selection by size filtering of 191k diffraction patterns and performing the EM algorithm on 18k patterns in the size range 55-84 nm. Size estimation takes 16 min 26s. It is single threaded and do not really benefit from many cores. Extraction and saving of filtered data take: 20 min 37 s. It is limited by storage read and write speed. EM classification takes 26 min 16 s for 10 iterations. For 5 classifications it is 2 h 11 min 20 s. Calculations were performed on a computer cluster node (max-exfl027) with 2 Intel E5-2698 v4 @ 2.20GHz. It is 40 cores and 80 threads total. The node also has 512GB of memory, but it is barely used by EM.

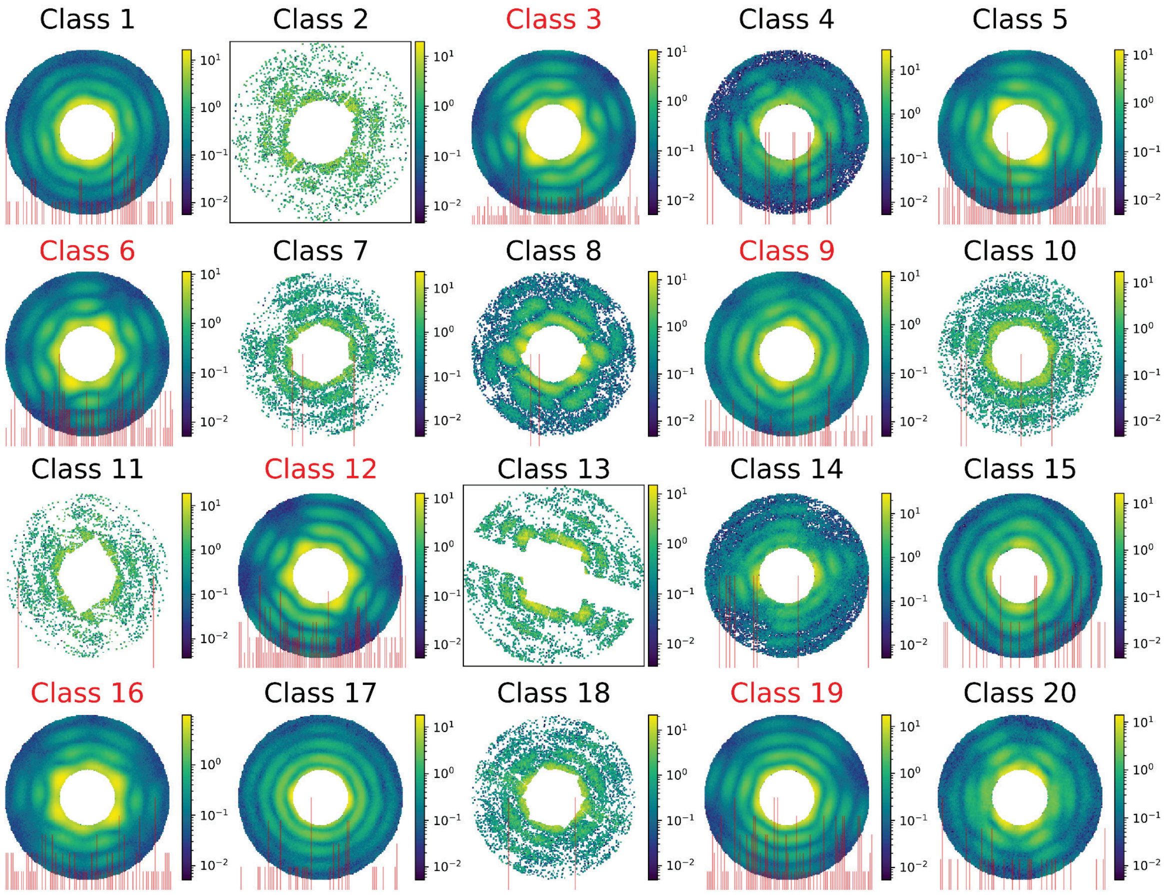

Figure S3 EM-based classification of single hit diffraction patterns for MaxF1 data set. Data were distributed into 20 classes, Classes 3, 6, 9, 12, 16, 19 were selected as containing diffraction patterns of PR772. These classes contain 893 patterns in total.

S6. Orientation determination results for different data selections

Orientation determination of the diffraction patterns was performed by using Expand-maximize-compress (EMC) algorithm (Loh & Elser, 2009) in the software Dragonfly (Ayyer *et al.*, 2016). The result of such a procedure is the 3D intensity distribution of the investigated particle (Fig. S4).

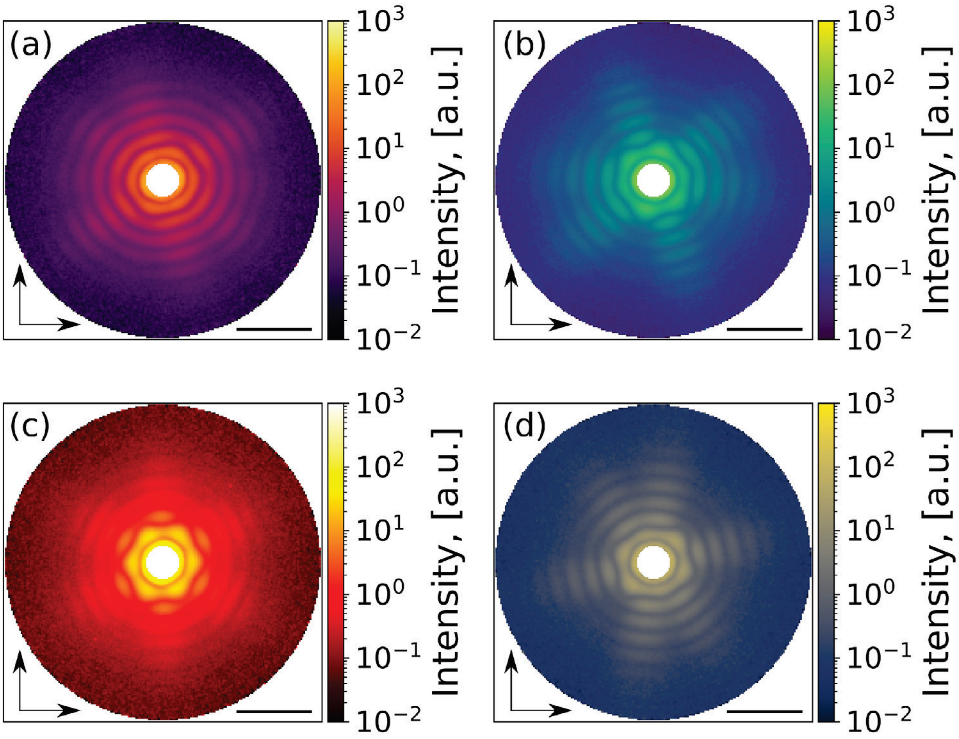

Figure S4 Result of orientation determination. 2D central slice of 3D intensity distribution for MaxF1 with the size filtering applied (a), MaxF1 with the EM algorithm and size filtering applied (b), moreSH with the size filtering applied (c), moreSH with the EM algorithm and size filtering applied (d). Black scale bar in denotes 0.5 nm^{-1}, vertical and horizontal axes denotes q_z and q_y directions, respectively.

Background level as the mean signal in the high q-region, was subtracted from each data set: MaxF1 with the size filtering applied, MaxF1 with the EM algorithm and size filtering applied, moreSH with the size filtering applied, moreSH with the EM algorithm and size filtering applied; results are shown in Fig. 6 and Fig. S5.

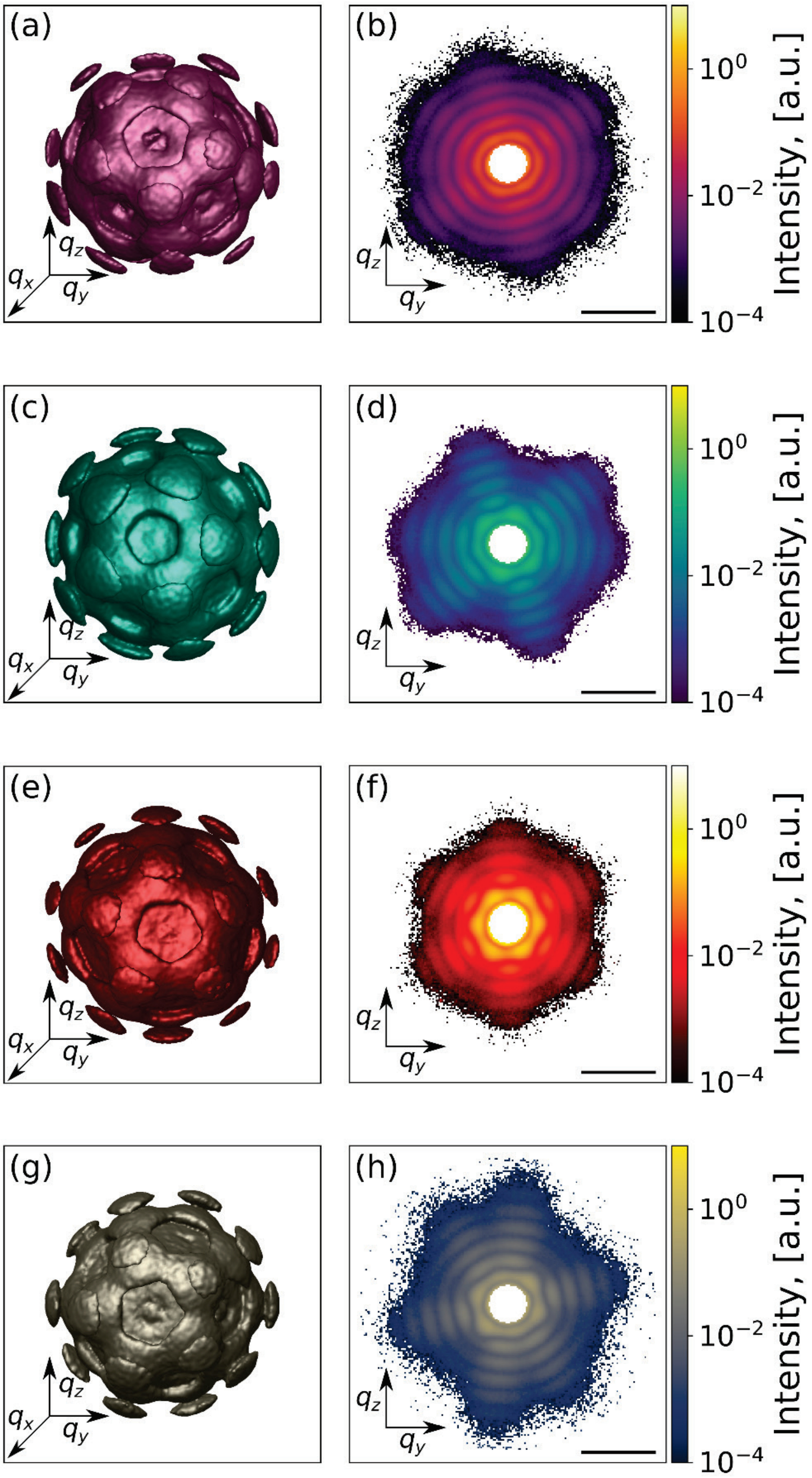

Figure S5 Reciprocal space representation for different data selections. (a,b) 3D intensity distribution and its 2D cut of MaxF1 with the size filtering applied data selection. (c,d) 3D intensity distribution and its 2D cut of moreSH with the size filtering applied. (e,f) 3D intensity distribution and

its 2D cut of moreSH with the EM algorithm and size filtering applied. All diffraction patterns are shown in logarithmic scale. Black scale bar in (a,d,f) denotes 0.5 nm^{-1}.

S7. Resolution

In order to numerically measure the difference between electron density reconstruction results from different data selections, we calculated Fourier-shell correlation (FSC) resolution (Harauz & van Heel, 1986). For the FSC-analysis the data set was divided into two sets, each of them oriented, reconstructed and then compared to each other. As for the resolution criterion, the 1/2-bit threshold (van Heel & Schatz, 2005) was used and is related to the signal-to-noise ratio of the two reconstructions. The resolution estimation is the intersection point of the 1/2-bit threshold with the FSC-curve.

Obtained FSC resolution for all four data sets (MaxF1 and moreSH with/without EM algorithm applied, with/without size filtering applied) fluctuates from 5.8 nm to 8 nm and is shown in Table S3 and Fig. S6. Applied EM algorithms for the CNN-based classification could improve the reconstruction result by several nanometers in terms of FSC resolution. And CNN-based single hit diffraction patterns classification by itself with size filtering applied could give quite good resolution. In comparison with the previous EM selection (Assalauova *et al.*, 2020) with 6.9 nm resolution, the results obtained in this work showed overall agreement in virus structure (Fig. 7) and FSC resolution, the difference varies +/- 1 nm. The best result appeared to be MaxF1 with the EM algorithm and the size filtering applied selection - with the FSC resolution of 5.8 nm. Corresponding inner structure (Fig. 7(c)) and 2D central slice (Fig. 7(d)) demonstrated only slight variance from the previous work (Assalauova *et al.*, 2020).

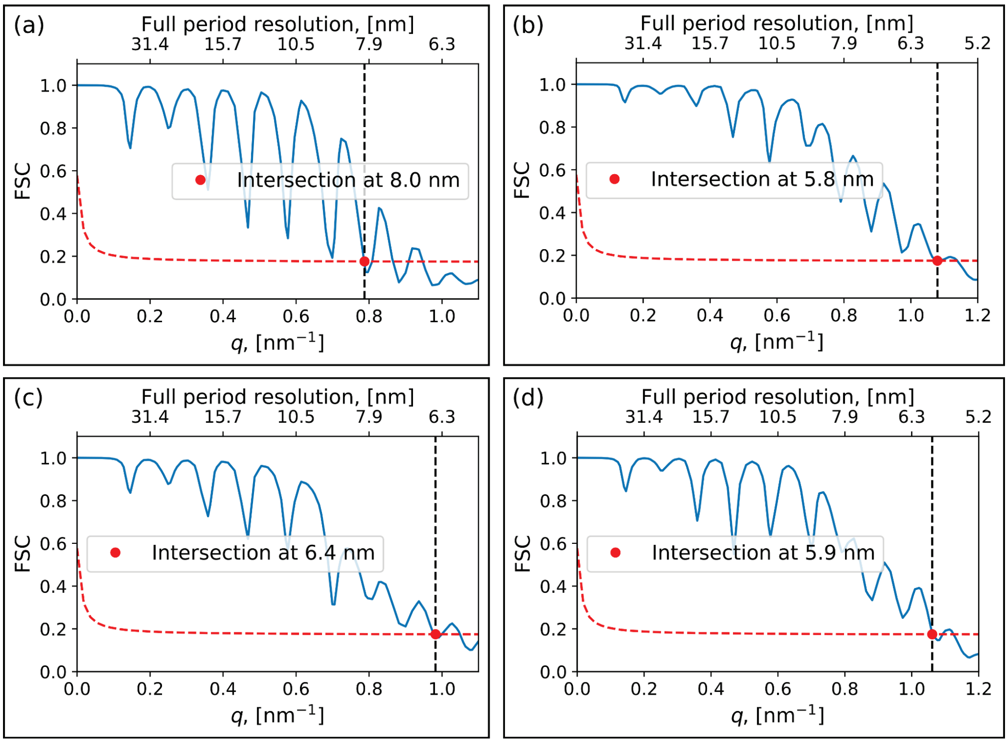

Figure S6 Fourier shell correlation resolution for different data selections. In all cases 1/2-bit threshold (red dashed line) was used. (a, b) Resolution for MaxF1 with size filtering applied (a) and MaxF1 with EM algorithm and size filtering applied (b). (c, d) Resolution for moreSH with size filtering applied (c) and moreSH with EM algorithm and size filtering applied (d).

Table S3 FSC resolution for different data selections.

**Data set**	**FSC resolution, nm**
MaxF1 + size selection	8
MaxF1 + EM + size selection	5.8
moreSH + size selection	6.4
moreSH + EM + size selection	5.9

S8. References

Aquila, A., Barty, A., Bostedt, C., Boutet, S., Carini, G., DePonte, D., Drell, P., Doniach, S., Downing, K. H., Earnest, T., Elmlund, H., Elser, V., Gühr, M., Hajdu, J., Hastings, J., Hau-Riege, S. P., Huang, Z., Lattman, E. E., Maia, F. R. N. C., Marchesini, S., Ourmazd, A., Pellegrini, C., Santra, R., Schlichting, I., Schroer, C., Spence, J. C. H., Vartanyants, I. A., Wakatsuki, S., Weis, W. I. & Williams, G. J. (2015). *Struct. Dyn.* **2**, 041701.

Assalauova, D., Kim, Y. Y., Bobkov, S., Khubbutdinov, R., Rose, M., Alvarez, R., Andreasson, J., Balaur, E., Contreras, A., DeMirci, H., Gelisio, L., Hajdu, J., Hunter, M. S., Kurta, R. P., Li, H., McFadden, M., Nazari, R., Schwander, P., Teslyuk, A., Walter, P., Xavier, P. L., Yoon, C. H., Zaare, S., Ilyin, V. A., Kirian, R. A., Hogue, B. G., Aquila, A. & Vartanyants, I. A. (2020). *IUCrJ*. **7**, 1102–1113.

Ayyer, K., Lan, T.-Y., Elser, V. & Loh, N. D. (2016). *J. Appl. Crystallogr.* **49**, 1320–1335.

Ayyer, K., Morgan, A. J., Aquila, A., DeMirci, H., Hogue, B. G., Kirian, R. A., Xavier, P. L., Yoon, C. H., Chapman, H. N. & Barty, A. (2019). *Opt. Express*. **27**, 37816.

Benner, W. H., Bogan, M. J., Rohner, U., Boutet, S., Woods, B. & Frank, M. (2008). *J. Aerosol Sci.* **39**, 917–928.

Chen, L.-C., Papandreou, G., Kokkinos, I., Murphy, K. & Yuille, A. L. (2018). *IEEE Trans. Pattern Anal. Mach. Intell.* **40**, 834–848.

Clark, J. N., Huang, X., Harder, R. & Robinson, I. K. (2012). *Nat. Commun.* **3**, 993.

Cruz-Chú, E. R., Hosseinizadeh, A., Mashayekhi, G., Fung, R., Ourmazd, A. & Schwander, P. (2021). *Struct. Dyn.* **8**, 014701.

Damiani, D., Dubrovin, M., Gaponenko, I., Kroeger, W., Lane, T. J., Mitra, A., O'Grady, C. P., Salnikov, A., Sanchez-Gonzalez, A., Schneider, D. & Yoon, C. H. (2016). *J. Appl. Crystallogr.* **49**, 672–679.

Dempster, A. P., Laird, N. M. & Rubin, D. B. (1977). *J. R. Stat. Soc. Ser. B*. **39**, 1–22.

DeVries, T. & Taylor, G. W. (2017). *ArXiv Prepr. ArXiv1708.04552*.

Ferguson, K. R., Bucher, M., Bozek, J. D., Carron, S., Castagna, J.-C., Coffee, R., Curiel, G. I., Holmes, M., Krzywinski, J., Messerschmidt, M., Minitti, M., Mitra, A., Moeller, S., Noonan, P., Osipov, T., Schorb, S., Swiggers, M., Wallace, A., Yin, J. & Bostedt, C. (2015). *J. Synchrotron Radiat.* **22**, 492–497.

Fienup, J. R. (1982). *Appl. Opt.* **21**, 2758.

Fienup, J. R. (2013). *Appl. Opt.* **52**, 45.

Gaffney, K. J. & Chapman, H. N. (2007). *Science (80-.).* **316**, 1444–1448.

Hantke, M. F., Hasse, D., Maia, F. R. N. C., Ekeberg, T., John, K., Svenda, M., Loh, N. D., Martin, A. V, Timneanu, N., Larsson, D. S. D., van der Schot, G., Carlsson, G. H., Ingelman, M., Andreasson, J., Westphal, D., Liang, M., Stellato, F., DePonte, D. P., Hartmann, R., Kimmel, N., Kirian, R. A., Seibert, M. M., Mühlig, K., Schorb, S., Ferguson, K., Bostedt, C., Carron, S., Bozek, J. D., Rolles, D., Rudenko, A., Epp, S., Chapman, H. N., Barty, A., Hajdu, J. &

Andersson, I. (2014). *Nat. Photonics*. **8**, 943–949.
Harauz, G. & van Heel, M. (1986). *Opt.* **73**, 146–156.
He, K., Zhang, X., Ren, S. & Sun, J. (2016). *European Conference on Computer Vision*, Vol. pp. 630–645. Springer.
van Heel, M. & Schatz, M. (2005). *J. Struct. Biol.* **151**, 250–262.
Ignatenko, A., Assalauova, D., Bobkov, S. A., Gelisio, L., Teslyuk, A. B., Ilyin, V. A. & Vartanyants, I. A. (2021). *Mach. Learn. Sci. Technol.* **2**, 025014.
Ioffe, S. & Szegedy, C. (2015). *International Conference on Machine Learning*, Vol. pp. 448–456. PMLR.
Isensee, F., Jaeger, P., Wasserthal, J., Zimmerer, D., Petersen, J., Kohl, S., Schock, J., Klein, A., RoSS, T. & Wirkert, S. (2020). *Zenodo Https://Doi. Org/10.5281/Zenodo*. **3632567**,.
Khubbutdinov, R., Menushenkov, A. P. & Vartanyants, I. A. (2019). *J. Synchrotron Radiat.* **26**, 1851–1862.
Kingma, D. P. & Ba, J. (2014). *ArXiv Prepr. ArXiv1412.6980.*
Krizhevsky, A., Sutskever, I. & Hinton, G. E. (2012). *Adv. Neural Inf. Process. Syst.* **25**, 1097–1105.
Li, H., Nazari, R., Abbey, B., Alvarez, R., Aquila, A., Ayyer, K., Barty, A., Berntsen, P., Bielecki, J., Pietrini, A., Bucher, M., Carini, G., Chapman, H. N., Contreras, A., Daurer, B. J., DeMirci, H., Flűckiger, L., Frank, M., Hajdu, J., Hantke, M. F., Hogue, B. G., Hosseinizadeh, A., Hunter, M. S., Jönsson, H. O., Kirian, R. A., Kurta, R. P., Loh, D., Maia, F. R. N. C., Mancuso, A. P., Morgan, A. J., McFadden, M., Muehlig, K., Munke, A., Reddy, H. K. N., Nettelblad, C., Ourmazd, A., Rose, M., Schwander, P., Marvin Seibert, M., Sellberg, J. A., Sierra, R. G., Sun, Z., Svenda, M., Vartanyants, I. A., Walter, P., Westphal, D., Williams, G., Xavier, P. L., Yoon, C. H. & Zaare, S. (2020). *Sci. Data*. **7**, 404.
Loh, N.-T. D. & Elser, V. (2009). *Phys. Rev. E*. **80**, 026705.
Long, J., Shelhamer, E. & Darrell, T. (2015). *Proceedings of the IEEE Conference on Computer Vision and Pattern Recognition*, Vol. pp. 3431–3440.
Lundholm, I. V, Sellberg, J. A., Ekeberg, T., Hantke, M. F., Okamoto, K., van der Schot, G., Andreasson, J., Barty, A., Bielecki, J., Bruza, P., Bucher, M., Carron, S., Daurer, B. J., Ferguson, K., Hasse, D., Krzywinski, J., Larsson, D. S. D., Morgan, A., Mühlig, K., Müller, M., Nettelblad, C., Pietrini, A., Reddy, H. K. N., Rupp, D., Sauppe, M., Seibert, M., Svenda, M., Swiggers, M., Timneanu, N., Ulmer, A., Westphal, D., Williams, G., Zani, A., Faigel, G., Chapman, H. N., Möller, T., Bostedt, C., Hajdu, J., Gorkhover, T. & Maia, F. R. N. C. (2018). *IUCrJ*. **5**, 531–541.
Marchesini, S. (2007). *Rev. Sci. Instrum.* **78**, 011301.
Marchesini, S., He, H., Chapman, H. N., Hau-Riege, S. P., Noy, A., Howells, M. R., Weierstall, U. & Spence, J. C. H. (2003). *Phys. Rev. B*. **68**, 140101.
Nazari, R., Zaare, S., Alvarez, R. C., Karpos, K., Engelman, T., Madsen, C., Nelson, G., Spence, J. C.

H., Weierstall, U., Adrian, R. J. & Kirian, R. A. (2020). *Opt. Express*. **28**, 21749.

Neutze, R., Wouts, R., van der Spoel, D., Weckert, E. & Hajdu, J. (2000). *Nature*. **406**, 752–757.

Osipov, T., Bostedt, C., Castagna, J.-C., Ferguson, K. R., Bucher, M., Montero, S. C., Swiggers, M. L., Obaid, R., Rolles, D., Rudenko, A., Bozek, J. D. & Berrah, N. (2018). *Rev. Sci. Instrum.* **89**, 035112.

Reddy, H. K. N., Yoon, C. H., Aquila, A., Awel, S., Ayyer, K., Barty, A., Berntsen, P., Bielecki, J., Bobkov, S., Bucher, M., Carini, G. A., Carron, S., Chapman, H., Daurer, B., DeMirci, H., Ekeberg, T., Fromme, P., Hajdu, J., Hanke, M. F., Hart, P., Hogue, B. G., Hosseinizadeh, A., Kim, Y., Kirian, R. A., Kurta, R. P., Larsson, D. S. D., Duane Loh, N., Maia, F. R. N. C., Mancuso, A. P., Mühlig, K., Munke, A., Nam, D., Nettelblad, C., Ourmazd, A., Rose, M., Schwander, P., Seibert, M., Sellberg, J. A., Song, C., Spence, J. C. H., Svenda, M., Van der Schot, G., Vartanyants, I. A., Williams, G. J. & Xavier, P. L. (2017). *Sci. Data*. **4**, 170079.

Rose, M., Bobkov, S., Ayyer, K., Kurta, R. P., Dzhigaev, D., Kim, Y. Y., Morgan, A. J., Yoon, C. H., Westphal, D., Bielecki, J., Sellberg, J. A., Williams, G., Maia, F. R. N. C., Yefanov, O. M., Ilyin, V., Mancuso, A. P., Chapman, H. N., Hogue, B. G., Aquila, A., Barty, A. & Vartanyants, I. A. (2018). *IUCrJ*. **5**, 727–736.

Scheres, S. H. W., Valle, M., Nuñez, R., Sorzano, C. O. S., Marabini, R., Herman, G. T. & Carazo, J.-M. (2005). *J. Mol. Biol.* **348**, 139–149.

Shi, Y., Yin, K., Tai, X., DeMirci, H., Hosseinizadeh, A., Hogue, B. G., Li, H., Ourmazd, A., Schwander, P., Vartanyants, I. A., Yoon, C. H., Aquila, A. & Liu, H. (2019). *IUCrJ*. **6**, 331–340.

Sobolev, E., Zolotarev, S., Giewekemeyer, K., Bielecki, J., Okamoto, K., Reddy, H. K. N., Andreasson, J., Ayyer, K., Barak, I., Bari, S., Barty, A., Bean, R., Bobkov, S., Chapman, H. N., Chojnowski, G., Daurer, B. J., Dörner, K., Ekeberg, T., Flückiger, L., Galzitskaya, O., Gelisio, L., Hauf, S., Hogue, B. G., Horke, D. A., Hosseinizadeh, A., Ilyin, V., Jung, C., Kim, C., Kim, Y., Kirian, R. A., Kirkwood, H., Kulyk, O., Küpper, J., Letrun, R., Loh, N. D., Lorenzen, K., Messerschmidt, M., Mühlig, K., Ourmazd, A., Raab, N., Rode, A. V., Rose, M., Round, A., Sato, T., Schubert, R., Schwander, P., Sellberg, J. A., Sikorski, M., Silenzi, A., Song, C., Spence, J. C. H., Stern, S., Sztuk-Dambietz, J., Teslyuk, A., Timneanu, N., Trebbin, M., Uetrecht, C., Weinhausen, B., Williams, G. J., Xavier, P. L., Xu, C., Vartanyants, I. A., Lamzin, V. S., Mancuso, A. & Maia, F. R. N. C. (2020). *Commun. Phys.* **3**, 97.

Strüder, L., Epp, S., Rolles, D., Hartmann, R., Holl, P., Lutz, G., Soltau, H., Eckart, R., Reich, C., Heinzinger, K., Thamm, C., Rudenko, A., Krasniqi, F., Kühnel, K.-U., Bauer, C., Schröter, C.-D., Moshammer, R., Techert, S., Miessner, D., Porro, M., Hälker, O., Meidinger, N., Kimmel, N., Andritschke, R., Schopper, F., Weidenspointner, G., Ziegler, A., Pietschner, D., Herrmann, S., Pietsch, U., Walenta, A., Leitenberger, W., Bostedt, C., Möller, T., Rupp, D., Adolph, M., Graafsma, H., Hirsemann, H., Gärtner, K., Richter, R., Foucar, L., Shoeman, R. L., Schlichting, I. & Ullrich, J. (2010). *Nucl. Instruments Methods Phys. Res. Sect. A Accel. Spectrometers,*

Detect. Assoc. Equip. **614**, 483–496.

Szegedy, C., Toshev, A. & Erhan, D. (2013). *NIPS.*

Wu, L., Juhas, P., Yoo, S. & Robinson, I. (2021). *IUCrJ.* **8**, 12–21.

Wu, L., Yoo, S., Suzana, A. F., Assefa, T. A., Diao, J., Harder, R. J., Cha, W. & Robinson, I. K. (2021). *Npj Comput. Mater.* **7**, 175.

Xu, B., Wang, N., Chen, T. & Li, M. (2015). *ArXiv Prepr. ArXiv1505.00853*.

Yang, X., Kahnt, M., Brückner, D., Schropp, A., Fam, Y., Becher, J., Grunwaldt, J.-D., Sheppard, T. L. & Schroer, C. G. (2020). *J. Synchrotron Radiat.* **27**, 486–493.

Zimmermann, J., Langbehn, B., Cucini, R., Di Fraia, M., Finetti, P., LaForge, A. C., Nishiyama, T., Ovcharenko, Y., Piseri, P., Plekan, O., Prince, K. C., Stienkemeier, F., Ueda, K., Callegari, C., Möller, T. & Rupp, D. (2019). *Phys. Rev. E.* **99**, 063309.
